# THE DYNAMIC RELATIONSHIP BETWEEN MULTIDIMENTIONAL ENVIRONMENT AND PHYSICAL ACTIVITY PARTICIPATION

**DOI:** 10.1093/geroni/igac059.2882

**Published:** 2022-12-20

**Authors:** Yalu Zhang, Donghui Mei, Heming Pei, Gong Chen

**Affiliations:** Peking University, Beijing, Beijing, China (People’s Republic); Peking University, Beijing, Beijing, China (People’s Republic); Peking University, Beijing, Beijing, China (People’s Republic); Peking University, Beijing, Beijing, China (People’s Republic)

## Abstract

**Objectives:**

Physical activity participation is a preventive factor in improving physical and cognitive functions. Both physical and social environments are essential in evaluating older adults’ opportunities to participate in physical activities. This study aims at assessing the multidimensional environment in physical activity participation and examining how the change in the multidimensional environments could affect their participation in physical activities.

**Methods:**

This paper combined the nationally representative data, the China Longitudinal Aging Social Survey data 2014–2018, and the county-level administrative data extracted from various statistical yearbooks. It used the latent variable cross-lagged panel model to capture the dynamic relationship between environment and physical activities. Additionally, the dynamic analytical model considers a multidimensional environment, including indoor-, outdoor-, human-, social-environment, etc., by adding the latent variables.

**Results:**

The descriptive statistics show that older adults who are in a better environment (.147 in the indoor environment, p < 0.001; .389 in the outdoor environment, p < 0.01; .571 in family support, p < 0.05; .346 in childhood activity experience, p < 0.001) are more likely to frequently participate in physical activities (three times or more per week). The results of the latent cross-lagged panel models indicate that every one percent increase in the multidimensional environmental index is expected to cause a 4.1% increase in the likelihood of participating in physical activities among Chinese older adults. Discussion: In addition to the continuous efforts to improve the physical environment, policymakers and researchers should pay more attention to the social environment to encourage more older adults to participate in physical activities.

